# Study of Bioengineered Zebra Fish Olfactory Receptor 131-2: Receptor Purification and Secondary Structure Analysis

**DOI:** 10.1371/journal.pone.0015027

**Published:** 2010-11-25

**Authors:** Kwong-Joo Leck, Shuguang Zhang, Charlotte A. E. Hauser

**Affiliations:** 1 Membrane Protein Nanobiotechnology Laboratory, Institute of Bioengineering and Nanotechnology, Singapore, Singapore; 2 Center for Biomedical Engineering, Massachusetts Institute of Technology, Cambridge, Massachusetts, United States of America; Universität Heidelberg, Germany

## Abstract

How fishes are able to detect trace molecules in large bodies of water is not understood. It is plausible that they use olfactory receptors to detect water-soluble compounds. How the zebra fish *Danio Rerio*, an organism with only 98 functional olfactory receptors, is able to selectively detect and recognize numerous compounds in water remains a puzzling phenomenon. We are interested in studying the biochemical and molecular mechanisms of olfaction in fish. Here, we report on the study of a bioengineered zebra fish olfactory receptor OR131-2, affinity-purified from a HEK293S tetracycline-inducible system. This receptor was expressed and translocated to the cell plasma membrane as revealed by confocal microscopy. Circular dichroism spectroscopy showed that the purified zebra fish receptor folded into an α-helical structure, as observed for other G-protein coupled receptors (GPCRs). Our study shows that it is possible to produce viable quantities of the zebra fish olfactory receptor. This will not only enable detailed structural and functional analyses, but also aid in the design of biosensor devices in order to detect water-soluble metabolites or its intermediates, which are associated with human health.

## Introduction

Membrane proteins play a number of vital roles in all living systems. Approximately 30% of all genes in almost all sequenced genomes code for membrane proteins [1], [2], [3]. However, our detailed understanding of their structures and functions lags far behind that of soluble proteins. As of September 2010, there are over 67,000 structures in the Protein Data Bank (http://www.rcsb.org/pdb/home/home.do), but only 686 membrane protein structures and only 254 of these are unique structures (http://blanco.biomol.uci.edu/Membrane_Proteins_xtal.html).

There are several bottlenecks in the study of membrane proteins. One of them is the need for an inexpensive method for large-scale production of soluble non-aggregated membrane proteins. This must be accompanied by systematic detergent screens to select detergents that are suitable for long-term stabilization of functional membrane proteins. Only then will it be possible to carry out successful crystallographic screens for structural analyses.

Olfaction is not only common in all animals, insects and microbes, but it is also a fascinating phenomenon of nature. We are interested in understanding how fish detect an extremely scarce food source in a large body of water. It is plausible that fish use olfactory receptors to detect water-soluble compounds. The critical question of how a zebra fish with only 98 functional olfactory receptors (and 35 pseudo-genes) [4], [5] is able to exclusively recognize countless compounds in water still remains a mystery. Olfactory receptors belong to the class A G-protein coupled receptor (GPCR) family, and they possess seven hydrophobic membrane spanning domains typical of all GPCRs [6], [7], [8]. It is extremely difficult to obtain large amounts of purified receptor proteins for biochemical and structural studies. Thus far, there are only 4 unique GPCR structures that have been solved and none of them are olfactory receptors [9], [10], [11], [12]. In order to fully understand how fish olfactory receptors recognize water-soluble compounds, it is crucial to obtain their detailed molecular structure, which will provide fundamental insights into how they function. Furthermore, we are interested in designing aqueous based sensing devices for a wide range of uses. A thorough understanding of fish olfaction will clearly support us in this task. Here, we report on three zebra fish olfactory receptor OR131-2 genes that were engineered for our current study. We purified zebra fish OR131-2 receptor proteins from HEK293S tetracycline-inducible cells stably expressing these OR131-2 receptor genes. Through confocal microscopy, we could demonstrate that the receptor was expressed in the plasma membrane. Circular dichroism spectroscopy showed that the purified zebra fish receptor contains α-helices, similar to the α-helical character demonstrated in other G-protein coupled receptors, suggesting that the purified protein folded correctly.

## Results

In order to carry out structural studies, a large amount of receptors is required. To accomplish this, we established a number of mammalian cell lines stably expressing the zebra fish OR131-2 receptor upon induction. We successfully selected a few stable OR131-2 receptor expressing cell lines after clonal selection. We have also optimized the growth and induction conditions of these cell lines for the production of OR131-2 proteins and have worked out a simple method to recover and purify the protein from cell lysates. Our investigations are described in detail below.

### Generation of OR131-2 Protein Expressing Cells

The method to produce milligram quantities of human olfactory receptors from HEK293S cells has previously been described [13], [14]. We have adopted a similar approach to produce OR131-2 protein expressing HEK293S cells. We used three different bioengineered constructs with the objective that at least one of them should be crystallized at a later time point for structural studies (Supplementary Figure S1): OR131-2C is the native receptor with a specific rhodopsin derived tag for affinity purification [15]. OR131-2A and OR131-2B have additional mutations at two potential N-glycosylation sites at the N-terminus of the protein where the cognate asparagine residues have been mutated to glutamine, in order to avoid any issues with heterogeneity in the glycosylation pattern interfering with subsequent structural analyses. Further, OR131-2B has a bacterial phage T4 lysozyme sequence bioengineered into the third intracellular loop to aid in later planned crystallization studies [11].

These genes were cloned into a tetracycline inducible vector, pcDNA4/To, which allows external control of protein expression. After transfection of genes into HEK293S cells, we monitored transgene expression in stably transduced cells by immunostaining with a rhodopsin 1D4 (Rho1D4) monoclonal antibody, which recognizes a 9 amino acid TETSQVAPA tag [15] bioengineered into the C-terminus of the OR131-2 receptor. As expected, the expression of OR131-2 receptor increases with the concentration of tetracycline in the culture media (Supplementary Figure S2). We found that the treatment of cells with 1 µg/mL tetracycline for 48 hours results in an optimal level of protein expression for most of the cell clones, without any significant toxic effects.

### Selection of OR131-2 Receptor Protein Expression Cell Clones

**Figure 1:**
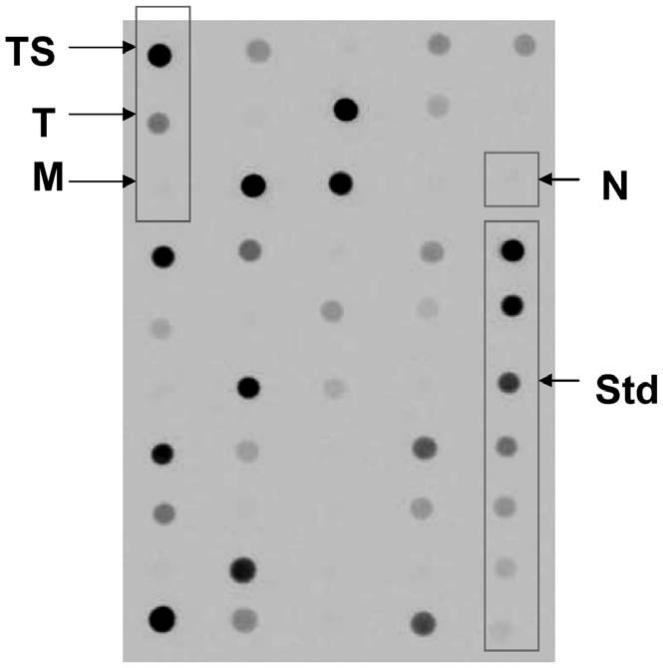
Dot blot screening of HEK293S cell clones using Rho1D4 monoclonal antibody. Cell lysates from HEK293S cell clones that have been treated with 1 µg/mL tetracycline and 2.5 mM sodium butyrate (TS), 1 µg/mL tetracycline only (T) or mock treated (M) for 48 hours were spotted onto a nitrocellulose membrane, and stained with a Rho1D4 monoclonal antibody. The Rho1D4 antibody specifically recognizes a nine amino acid TETSQVAPA tag on the carboxyl terminus of recombinant zebra fish OR131-2 protein. Serial two fold dilutions of purified TETSQVAPA tagged human OR17-4 protein (50 ng/ml –0.78 ng/ml) were spotted as positive control standards (Std) to estimate protein yield, and identically prepared cell lysates from HEK293S cells (N) served as negative controls. Altogether, more than 200 HEK293S cell clones expressing the OR131-2 proteins were screened and the higher producing cell clones were selected for further screening to examine the quality of the protein produced.

**Figure 2:**
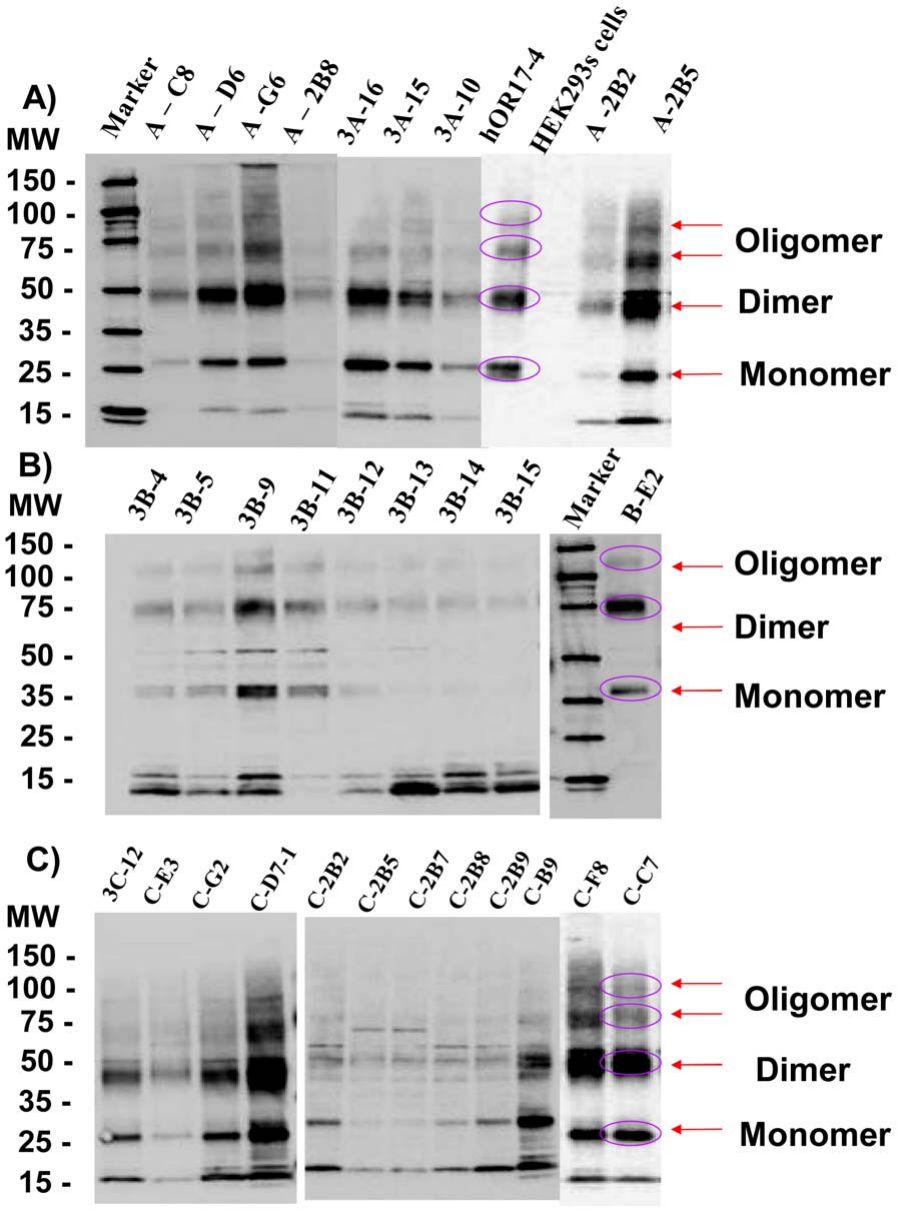
Western blot analyses of selected HEK293s cell clones expressing OR131-2 proteins after 48 hours treatment with 1 µg/mL tetracycline and 2.5 mM sodium butyrate. Fos-Choline14 soluble proteins isolated from harvested cells, were separated on Novex® 4–12% Bis-Tris gels, blotted on Polyvinylidene Fluoride (PVDF) membranes and stained with Rho1D4 monoclonal antibody. A) HEK293s cell clones expressing OR131-2A with protein sequence shown in supplementary Figure 1. Purified Rho1D4-tagged human olfactory receptor 17-4 (hOR17-4) solubilized in Fos-Choline 14 was loaded in gel as positive controls while the same concentration of Fos-Choline dissolved cell lysates from un-transfected HEK293s cells served as negative control. Similar to purified hOR17-4, OR131-2 proteins migrated faster on SDS gels than what their theoretical molecular weights predict. Monomeric, dimeric and oligomeric forms of the proteins were visible on the gel. B) HEK293s cell clones expressing OR131-2B with protein sequence as shown in supplementary Figure 1. The insertion of T4-lysozyme in the third intracellular loop of OR131-2 results in an expected gel shift of the protein monomer, dimer and oligomers. C) HEK293s cell clones expressing OR131-2C with sequence shown in supplementary Figure S1.

It has been reported that sodium butyrate, an inhibitor of histone deacetylase, can frequently be added to culture media to enhance recombinant protein expression in mammalian cells [16]. We tested whether the addition of sodium butyrate to the culture media would further augment protein expression in cells already induced with tetracycline. Zeocin resistant cell clones were mock-treated (negative control), treated with tetracycline or treated with both tetracycline and sodium butyrate to induce OR131-2 protein expression. We evaluated the efficiency of protein expression in different cell clones using immunofluorescence staining (Supplementary Figure S2) and dot-blot analysis (Figure 1). We found that a combination of 1 µg/mL tetracycline and 2.5 mM sodium butyrate induced the highest level of protein expression in most cell clones after 48 hours. From more than 200 cell clones screened by dot blot analysis, cell lysates from 30 of the most productive clones were further analyzed by western blot analysis to examine the quality of the protein produced (Figure 2). The human olfactory receptor 17-4 (hOR17-4) [17] was previously purified [13], [14] and used in some of the gels as positive controls, since this olfactory receptor share a similar molecular weight and a common seven trans-membrane (7-TM) structure. Similar to the hOR17-4 and other membrane proteins, the OR131-2 proteins migrated on SDS-PAGE faster than predicted by their theoretical molecular weights [18]. We could observe what appeared to be the monomeric, dimeric and higher molecular weight oligomeric forms of the protein. OR131-2A and OR131-2C, which have molecular weights similar as hOR17-4, migrated in a similar fashion to purified hOR17-4 (Figure 2). OR131-2B, which has a 160 amino acid insert from T4 lysozyme in its third intracellular loop, showed a shift in molecular weight of about 20 kDa (Figure 2B). The highest odorant receptor protein producing cell clones judged by Western blot analyses were selected for scale-up protein production and subsequent purification experiments.

### Purification of OR131-2 Proteins

**Figure 3:**
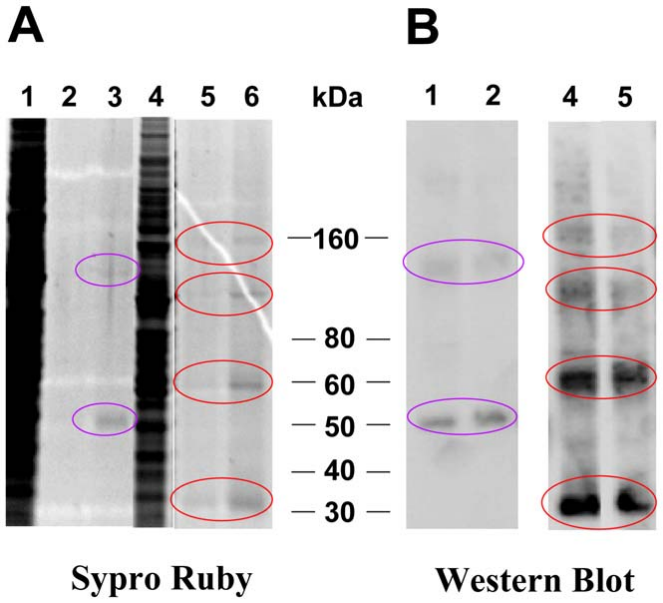
Purification of OR131-2 proteins with Rho1D4 monoclonal antibody conjugated sepharose 4B beads. Purified Fos-Choline 14 solubilized proteins were separated on Novex® 4–12% Bis-Tris gels and either A) stained with Sypro Ruby protein stain or B) blotted on Polyvinylidene Fluoride (PVDF) membrane and stained with Rho1D4 monoclonal antibody. Shown here are the purification of two independent protein clones with protein sequences A and B (see supplementary Figure S1). Lane 1: Crude protein lysate from cells expressing OR131-2B. Lane 2: purified OR131-2B eluent (in the same volume of buffer as initial crude protein lysate). Lane 3: Concentrate of purified OR131-2B eluent using Vivaspin 6 with a 10,000 molecular weight cut off. Lane 4: Crude protein lysate from cells expressing OR131-2A. Lane 5: purified OR131-2A eluent (in the same volume of buffer as initial crude protein lysate). Lane 6: Concentrate of purified OR131-2A eluent using Vivaspin 6 with a 10,000 molecular weight cut off.

Purification and stabilization of membrane proteins remains one of the most difficult challenges in producing high quality proteins for structural studies. Using immunoaffinity purification in a single purification step, we were able to obtain ∼290 µg purified protein per gram of culture cells, with protein purity of more than 85% and a 90% recovery rate. The Sypro Ruby protein stain in Figure 3A shows the purification of proteins from two representative cell clones. Figure 3B exhibits the Western blot results of the same cell clones where the protein eluent has approximately the same concentration of OR131-2 protein as the original crude cell lysate in the same solution volume. We have performed mass spectrometric analysis of the purified proteins and the results confirmed that the proteins are indeed OR131-2 (Supplementary Figure S3). In order to separate the monomer from its dimeric form, a further purification step via gel filtration could be used.

### Localization of OR131-2 Proteins in HEK293S Cells

**Figure 4:**
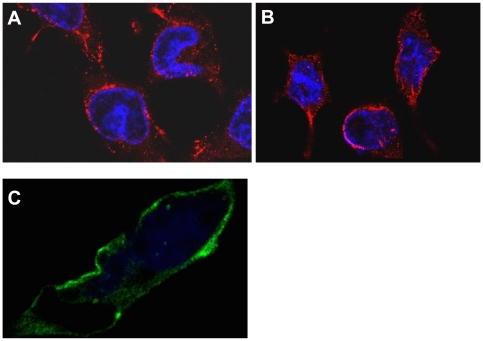
Confocal micrographs of OR131-2 expressing HEK293s cells. The OR131-2 protein expressed in HEK293 cells were immunofluorescently labelled with a Rho1D4 monoclonal antibody, followed by a species specific Tetramethylrhodamine (TRITC) labeled secondary antibody (panels A and B) or an Alexa Fluor 488 labeled secondary antibody (panel C). The cell nuclei were counter-stained stained with DAPI (4′,6-diamidino-2-phenylindole).

We used confocal microscopy to examine the HEK293S cells that expressed OR131-2 receptors in order to verify if the expressed OR131-2 receptor is correctly translocated into the cell plasma membrane. Figure 4 shows that the OR131-2 protein is indeed located in the cell membrane and also in the cytoplasmic region. Given the high over-expression level of OR131-2 receptors, it is expected that some of these receptors would be localized in intracellular membrane compartments, particularly in the endoplasmic reticulum and the Golgi apparatus. The number of expressed receptors also likely exceeds the actual receptor density of this particular receptor type on olfactory epithelial cells.

### Secondary Structural Analysis of the OR131-2 Receptor

**Figure 5:**
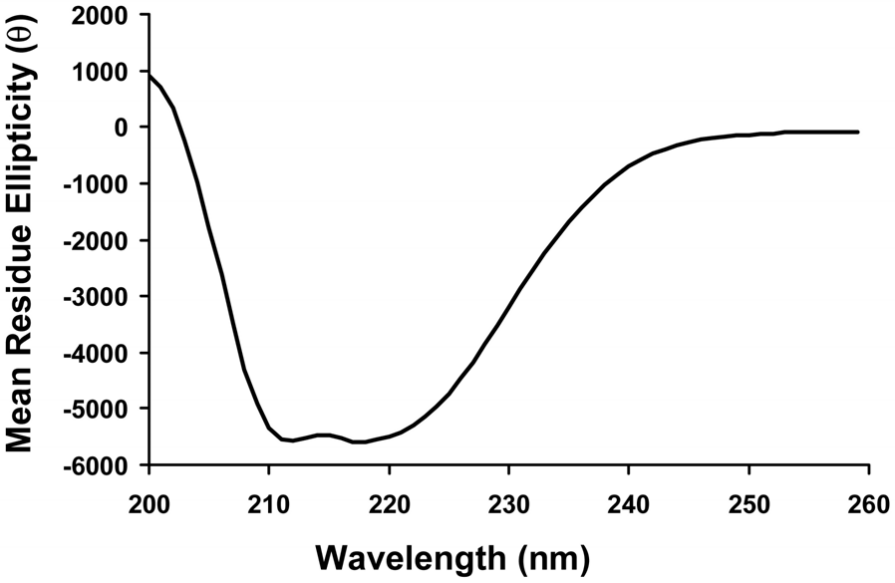
Secondary structural analysis of purified OR131-2 protein. The circular dichroism (CD) spectrum shows the presence of α-helical content in purified OR131-2 protein (0.87 mg/mL). Mean residue ellipticity [θ] has units millidegrees x cm^2^ x dmol^−1^.

In order to exert its biological function, the correct folding of a protein is crucial. We wanted to ensure that the purified OR131-2 receptor was correctly folded. Thus, we used circular dichroism (CD) analysis to examine the purified receptor for its α-helical character since almost all GPCRs are known to fold into seven trans-membrane helices [19]. For the fish OR131-2 receptor, this should be no exception. The CD spectrum of OR131-2 receptor is shown in figure 5. Analysis of our CD data using the CDNN software [20] revealed an alpha helical content of ∼30–35%, which was close to the percentage (∼43%) of trans-membrane helices predicted in OR131-2 with a bioinformatics software (http://www.cbs.dtu.dk/services/TMHMM-2.0/). The other major contributors to the CD spectrum came from random coils (∼25–30%) and beta turns (∼15–20%).

## Discussion

This study describes the purification of milligram quantities of zebra fish olfactory receptor 131-2 from HEK293S cells. The protein is correctly localized in cell membranes and CD analysis of the purified protein indicates the presence of alpha helical content, affirming our assumption that the purified protein is correctly folded. These results indicate the possibility that the simple GPCRs production and purification protocol described here may be generally applicable to other GPCRs, overcoming one of the first bottlenecks frequently encountered during structural studies of GPCRs.

Although circular dichroism spectroscopy is a useful technique in the determination of protein secondary structures [21], the reference databases used by various CD data analysis algorithms (including the CDNN used here) are derived from soluble proteins. Due to challenges in large scale production and purification of membrane proteins, CD spectra of membrane proteins are still relatively rare and it is debatable whether a database of reference spectra based on soluble proteins can be validly applied to membrane proteins [22], [23]. The work described here will eventually contribute to a reference database for membrane proteins.

Olfaction, in particular the mechanism of odorant binding, still remains a vastly unexplored area, although the further neuronal processing of olfaction after odorant receptor activation is a better understood process [24], [25], [26]. Furthermore, the sequenced genomes of many species reveal that the numbers of functional olfactory receptor and pseudo-genes vary widely. For example, dogs have 1,070 olfactory receptors [27], mice have 1,200 [28], rats have 1,430 [29], nematode worms have 1,100 [30], frogs have 410 [5], chimpanzees have 450 [31], humans have 388 [32], chickens have 78 [5], mosquitoes have 79 [33], Drosophila have 62 [34], puffer fishes have 44 [5] and zebra fishes have 98 [5] olfactory receptors.

Although zebra fish has only 98 olfactory receptors, it can distinguish a wide range of water-soluble compounds, pheromones and other molecules including amino acids, trace amines and decays of scarce food sources in extremely diluted concentrations. Since the zebra fish is an excellent genetic model of vertebrates with few olfactory receptors compared to other species, its mechanism of olfaction should be more easy to study as compared to more complex olfactory systems.

Currently, little is known about how fish olfactory receptors recognize odorants and other molecules in water. In other words, the OR131-2 receptor is still an orphan receptor without known ligands. It is possible that it could recognize more than one compound and perhaps many more compounds in a combinatorial manner as suggested for other olfactory receptors [24], [25], [26]. Experiments are underway to systematically identify its possible ligands.

OR131-2 is probably the first fish olfactory receptor that has been expressed, produced and purified to near-homogeneity for biochemical and structural studies. Our study may stimulate further studies of aqueous olfactory and chemosensory receptors from other amazing marine animals in deep oceans.

## Materials and Methods

### Materials

Fos-Choline14 (FC14) was purchased from Anatrace (Maumee, OH). All tissue culture media and media additives were purchased from Invitrogen Pte Ltd (Singapore) unless otherwise stated. Restriction enzymes were bought from Fermentas (Ontario, Canada). Sodium butyrate and Dulbecco's Phosphate Buffered Saline (D-PBS) were purchased from Sigma (St. Louis, MO). HEK293s cells stably transduced with pcDNA6/TR were a gift from Brian Cook of Massachusetts Institute of Technology, USA. Rho1D4 monoclonal antibody was purchased from Cell Essentials (Boston, MA, USA). The buffers used for protein purification are as follows: Wash buffer: D-PBS containing 0.2% wt/vol FC14; Elution buffer: Wash buffer containing 500 µM Ac-TETSQVAPA-CONH_2_ (First Base, Singapore) elution peptide.

### Generation of OR131-2 Protein Expressing Cells

HEK293S cells were cultured in Advanced Dulbecco's Modified Eagle Medium/F12 mixture supplemented with non-essential amino acids, 10% fetal bovine serum, 110 mg/mL sodium pyruvate, 15 mM Hepes, 100 units/mL penicillin, 100 µg/mL streptomycin and 5 µg/mL Blasticidin S. Transfection of genes into HEK293s cells was performed using Lipofectamine 2000 (Invitrogen, Singapore) following the manufacturer's instructions. Cell clones stably integrating the transgenes were selected over 3 weeks by supplementing the culture media with 250 µg/mL zeocin.

### Dot Blot Screening of Cell Clones

Zeocin resistant cell clones were either mock treated or treated with 1 µg/mL tetracycline, 1 µg/mL tetracycline and 2.5 mM sodium butyrate for 48 hours to induce OR131-2 protein expression. Cell lysates was prepared in D-PBS buffer containing protease inhibitor cocktail set III (Merck KGaA, Darmstadt, Germany) and 2% wt/vol FC14. One µL of cell lysate from each treatment condition was spotted onto nitrocellulose membrane, air dried for 30 minutes, incubated with Rho1D4 monoclonal antibody for 1 hour, followed by detection using the ECL Plus kit (GE Healthcare, Singapore) according to manufacturer's instructions.

### Immunoblotting and Total Protein Staining

Proteins in cell lysates were separated via sodium dodecyl sulfate**-**polyacrylamide gel electrophoresis (SDS-PAGE) on Novex® 4-12% Bis Tris gels (Invitrogen, Singapore). For immunoblotting, proteins were transferred to polyvinylidene fluoride (PVDF) membrane using a semi-dry transfer apparatus (BioRad, Singapore) or an I-Blot (Invitrogen, Singapore) according to instructions in the manufacturer's manual. The blot was blocked with 5% skim milk (BioRad, Singapore) and incubated with Rho1D4 monoclonal antibody for 2 hours, followed by signal detection using the ECL Plus kit as described above. Total protein staining was performed using the Sypro® Ruby protein gel staining kit (Invitrogen, Singapore) following the protocol described in the manual.

### Immunoaffinity Purification

Immunoaffinity purification was performed using Cyanogen Bromide-activated Sepharose 4B beads (GE Healthcare, Singapore) chemically cross-linked to Rho1D4 monoclonal antibody. To begin purification, cell lysates was incubated at 4°C overnight with the antibody conjugated beads, followed by washing the beads approximately 10 times with 4 bead volumes of wash buffer each time. OR131-2 proteins were eluted with elution buffer containing 500 µM Ac-TETSQVAPA-CONH_2_ elution peptide. Finally, the protein was concentrated and the elution peptides removed using a centrifugal concentrator with 10,000 molecular weight cut off, Vivaspin 6 (Sartorius Stedim Biotech SA, Aubagne Cedex, France) and buffer exchange was performed 10 times to remove the peptide.

### Confocal Microscopy

OR131-2 protein expression was induced in zeocin resistant cells cultured on poly L-Lysine (Sigma, St. Louis, MO) treated glass cover-slips by supplementing the culture media with 1 µg/mL tetracycline and 2.5 mM sodium butyrate for 48 hours. The culture media was removed and the cells were fixed and permeablized with ice cold methanol for 2 minutes. Methanol was completely removed by washing with Tris Buffered Saline (TBS) 3 times. To prevent non-specific binding, the cells were incubated in 3% wt/vol Bovine Serum Albumin (PAA Laboratories GmbH, Pasching, Austria) in TBS for 30 minutes. The cells were then stained with Rho1D4 monoclonal antibody diluted 1∶1000 in the above blocking buffer for 1 hour. After washing for 3 times with 0.1% Tween 20 (Sigma, St. Louis, MO) in TBS (TBST), cells were incubated with either a mouse specific Tetramethylrhodamine (TRITC) labeled secondary antibody (Sigma, St. Louis, MO) or an Alexa Fluor 488 labeled secondary antibody (Invitrogen, Singapore) for 1 hour. Following 5 washes with TBST to remove any non-specifically bound antibody, cells were counter-stained with the nucleus specific dye, 4′,6-diamidino-2-phenylindole (DAPI) (Sigma, St Louis, MO) for 5 minutes. Next, cover slips were removed from the wells and mounted on a microscopic glass slide using Hydromount (National Diagnostics, Atlanta, GA). Confocal imaging was performed on a Carl Zeiss LSM 510 META Upright microscope using a 100x oil objective.

### Circular Dichroism Spectroscopy

CD spectra were measured at 25°C over the wavelength range of 200–250 nm with a step size of 1 nm and an averaging time of 10 sec, using a CD spectrometer (Aviv Associates, Model 410). All spectra were the average of 5 replicate scans. Spectra shown for purified OR131-2 protein were baseline subtracted with wash buffer to remove the effects of the FC14 detergent. The measurements are reported in mean residue ellipticity (millidegrees x cm^2^ x dmole^−1^). Analyses of the CD spectra were performed using the CDNN software provided with the Aviv instrument. Protein concentration was determined using the BCA Protein Assay kit (Pierce Biotechnology, Rockford, IL).

## Supporting Information

Figure S1 A) Three different zebra fish olfactory receptor 131-2 constructs were made (Method S1) according to the modifications depicted in the table. OR131-2A contains Rho1D4 and Strep- Tactin purification tags on its C and N termini respectively and two potential N-glycosylation sites at the N terminus of the protein were mutated to glutamine. OR131-2B contains all the modifications described for OR131-2A and the 2-161 amino acid residues of T4-Lysozyme within its predicted third intracellular loop. OR13-2C is the native form of the protein with the Rho1D4 and Strep-Tactin purification tags. (B) Amino acid sequences of OR131-2 proteins. Modifications made to the native OR131-2 protein are underlined. These modifications include the addition of strep-tactin (WSHPQFEKQ) and Rho1D4 (TETSQVAPA) purification tags to the N and C terminus of the proteins respectively, the insertion of the 2-161 amino acid residues of bacteriophage T4 lysozyme in OR131-2B and mutations of two potential N glycosylation sites (highlighted in red) in OR131-2A and OR131-2B.
(TIF)

Figure S2 Immunofluorescence staining was performed as described (Method S2). Protein expression was induced in two representative cell clones by supplementing the culture media with tetracycline (0.1 to 1 µg/mL) for 48 hours. Non-induced cells served as negative controls. OR131-2 protein expression was detected by staining with a Rho1D4 monoclonal antibody, followed by a species specific tetramethylrhodamine (TRITC) labeled secondary antibody. The further addition of 2.5 mM sodium butyrate to culture media containing 1 µg/mL tetracycline leads to enhancement of protein expression in both cell clones.
(TIF)

Figure S3 Mass spectrometry is performed following standard procedure as described in Method S3. (A) Map of peptide fragments identified by LC-MS/MS showing extent of coverage. Extensive protein blast searches were performed on http://blast.ncbi.nlm.nih.gov and the blast results show that these peptide fragments uniquely identifies OR131-2. (B) Table of trypsin digested peptide sequences identified. (C) Mass spectrum of an identified peptide fragment YYAFCGIYVYK.
(TIF)

Method S1 Method for Supplementary Figure S1. Gene design strategies.
(DOC)

Method S2 Method for Supplementary Figure S2. Immunofluorescence staining.
(DOC)

Method S3 Method for Supplementary Figure S3. Mass Spectrometry.
(DOC)
