# Supplementary figures and images for: Study of Bioengineered Zebra Fish Olfactory Receptor 131-2: Receptor Purification and Secondary Structure Analysis
Source: PLoS One. 2010 Nov 25;5(11):e15027. doi: 10.1371/journal.pone.0015027 (PMC2993934; doi:10.1371/journal.pone.0015027)

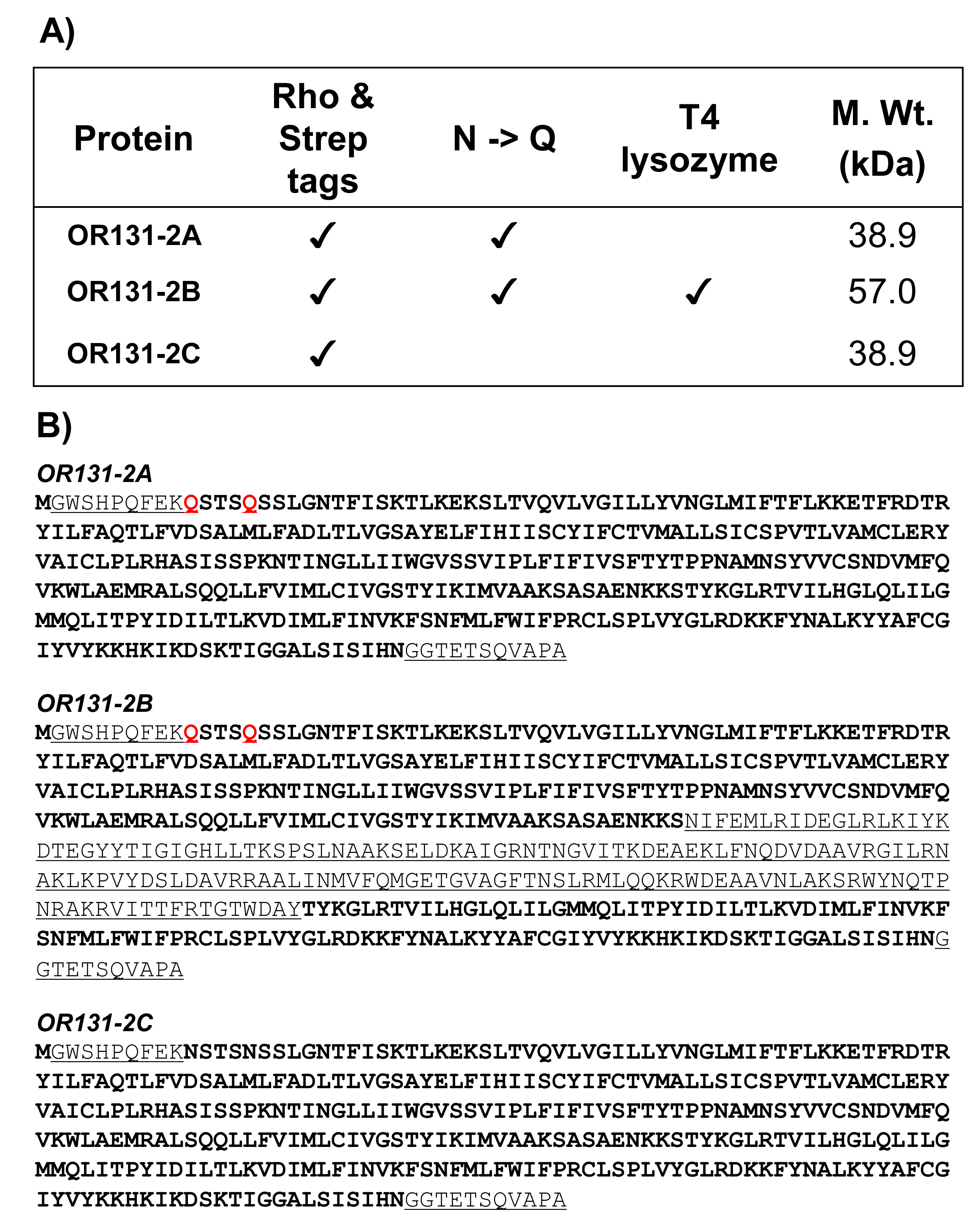

Supplement: Figure S1 — A) Three different zebra fish olfactory receptor 131-2 constructs were made (Method S1) according to the modifications depicted in the table. OR131-2A contains Rho1D4 and Strep- Tactin purification tags on its C and N termini respectively and two potential N-glycosylation sites at the N terminus of the protein were mutated to glutamine. OR131-2B contains all the modifications described for OR131-2A and the 2-161 amino acid residues of T4-Lysozyme within its predicted third intracellular loop. OR13-2C is the native form of the protein with the Rho1D4 and Strep-Tactin purification tags. (B) Amino acid sequences of OR131-2 proteins. Modifications made to the native OR131-2 protein are underlined. These modifications include the addition of strep-tactin (WSHPQFEKQ) and Rho1D4 (TETSQVAPA) purification tags to the N and C terminus of the proteins respectively, the insertion of the 2-161 amino acid residues of bacteriophage T4 lysozyme in OR131-2B and mutations of two potential N glycosylation sites (highlighted in red) in OR131-2A and OR131-2B. (TIF) [file pone.0015027.s001.tif]

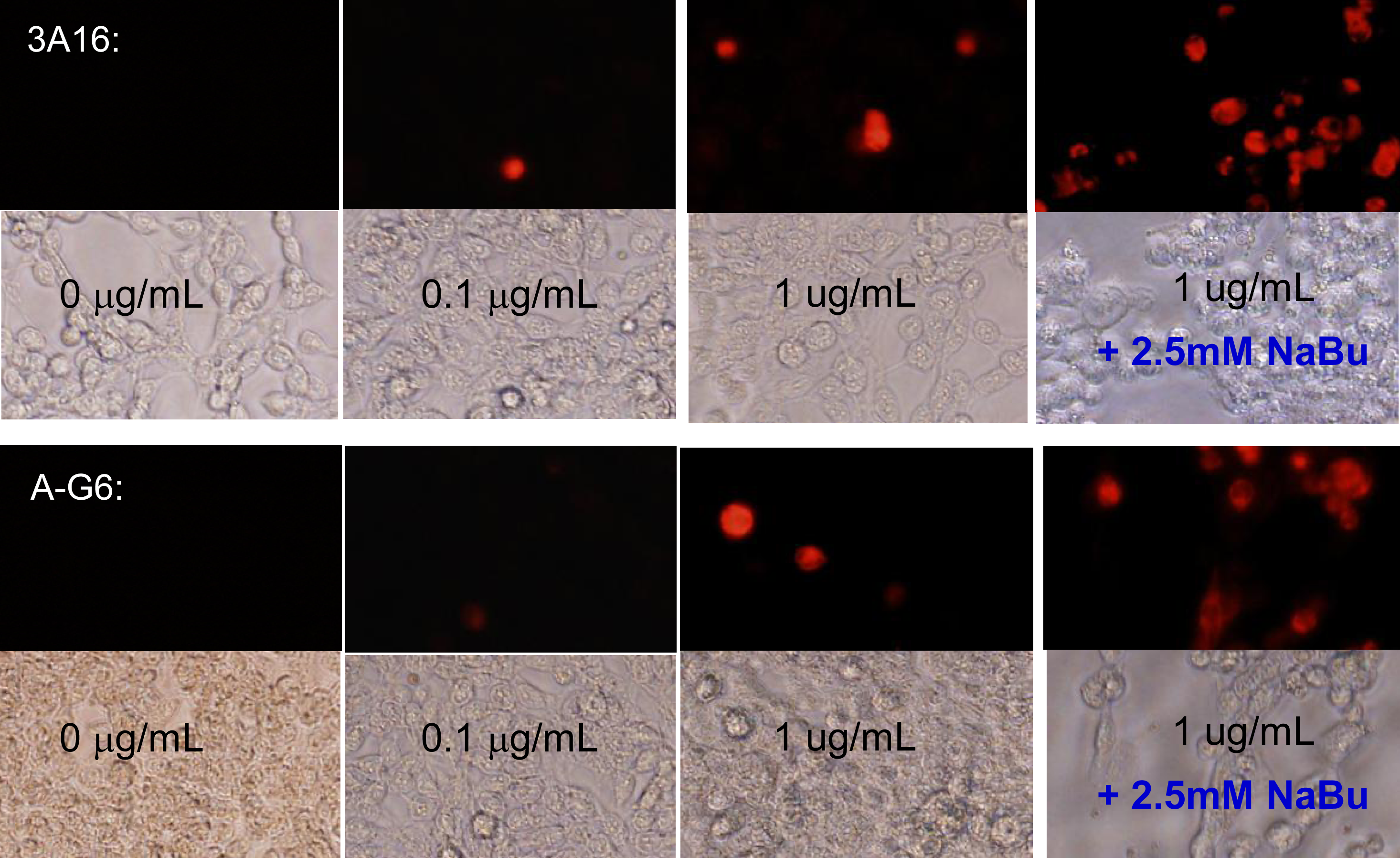

Supplement: Figure S2 — Immunofluorescence staining was performed as described (Method S2). Protein expression was induced in two representative cell clones by supplementing the culture media with tetracycline (0.1 to 1 µg/mL) for 48 hours. Non-induced cells served as negative controls. OR131-2 protein expression was detected by staining with a Rho1D4 monoclonal antibody, followed by a species specific tetramethylrhodamine (TRITC) labeled secondary antibody. The further addition of 2.5 mM sodium butyrate to culture media containing 1 µg/mL tetracycline leads to enhancement of protein expression in both cell clones. (TIF) [file pone.0015027.s002.tif]

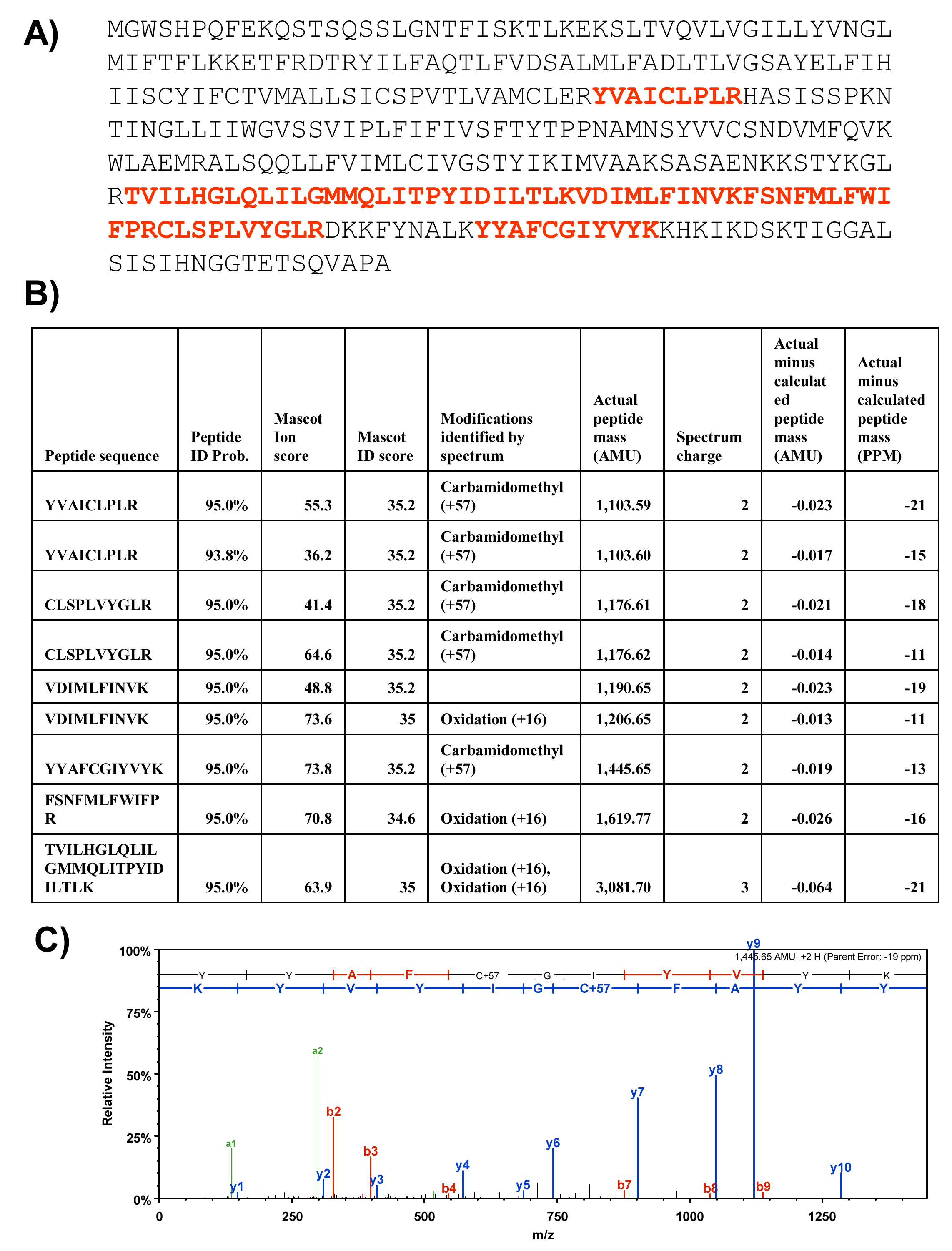

Supplement: Figure S3 — Mass spectrometry is performed following standard procedure as described in Method S3. (A) Map of peptide fragments identified by LC-MS/MS showing extent of coverage. Extensive protein blast searches were performed on http://blast.ncbi.nlm.nih.gov and the blast results show that these peptide fragments uniquely identifies OR131-2. (B) Table of trypsin digested peptide sequences identified. (C) Mass spectrum of an identified peptide fragment YYAFCGIYVYK. (TIF) [file pone.0015027.s003.tif]
