# Supplementary material for: Study of Bioengineered Zebra Fish Olfactory Receptor 131-2: Receptor Purification and Secondary Structure Analysis
Source: PLoS One. 2010 Nov 25;5(11):e15027. doi: 10.1371/journal.pone.0015027 (PMC2993934; doi:10.1371/journal.pone.0015027)
Supplement: Method S1 — Method for Supplementary Figure S1. Gene design strategies. (DOC) [file pone.0015027.s004.doc]

**Supplementary Method S1**

Gene Design

The 326 amino acids zebra fish Olfactory Receptor 131-2 (OR131-2) sequence was obtained directly from GenBank (<http://www.ncbi.nlm.nih.gov/protein/NP_001034727>). The location of trans-membrane helices was predicted using a bioinformatic software (<http://www.cbs.dtu.dk/services/TMHMM-2.0/>). A Strep-Tag (WSHPQFEK) and a Rho1D4 Tag (TETSQVAPA) with glycine spacers were added to the N- and C-terminus of the protein, respectively. These tags are useful for affinity purification of the protein. Potential N-glycosylation sites were identified with the help of a bioinformatics program (<http://www.cbs.dtu.dk/services/NetNGlyc/>). Altogether, three protein sequences were designed: OR131-2A, OR131-2B and OR131-2C (Supplementary Figure S1). OR131-2B contains a 160 amino acid insert from bacteriophage T4 lysozyme in the predicted third intracellular loop. The protein sequences were reverse-translated into DNA code and codon optimized for expression in human cells. Codon optimization and DNA synthesis were performed by GeneArt AG (Regensburg, Germany). The genes were subcloned into the EcoRI and XhoI sites of pcDNA4/To (Invitrogen, Singapore).
