# Supplementary material for: Study of Bioengineered Zebra Fish Olfactory Receptor 131-2: Receptor Purification and Secondary Structure Analysis
Source: PLoS One. 2010 Nov 25;5(11):e15027. doi: 10.1371/journal.pone.0015027 (PMC2993934; doi:10.1371/journal.pone.0015027)
Supplement: Method S2 — Method for Supplementary Figure S2. Immunofluorescence staining. (DOC) [file pone.0015027.s005.doc]

**Supplementary Method S2**

Immunofluorescence Staining

OR131-2 protein expressing cells were fixed and permeablized with ice cold methanol for 2 minutes, followed by 3 washes in Tris buffered saline (TBS) to completely remove methanol. Next, blocking buffer containing 3% wt/vol Bovine Serum Albumin (PAA Laboratories GmbH, Pasching, Austria) was applied for 30 minutes. The cells were then stained with Rho1D4 monoclonal antibody diluted 1:1000 in blocking buffer for 1 hour. After 3 washes with 0.1% Tween 20 (Sigma, St. Louis, MO) in TBS (TBST), cells were incubated with a Tetramethylrhodamine (TRITC) labeled anti-mouse IgG secondary antibody (Sigma, St. Louis, MO) for 1 hour. Following 5 washes with TBST to remove any non-specifically bound antibody, cells were counter-stained with the nucleus specific dye, 4',6-diamidino-2-phenylindole (DAPI) (Sigma, St Louis, MO) for 5 minutes. Next, the DAPI solution was replaced with 10% glycerol in TBS and the cells were visualized on an Olympus IX71 fluorescent microscope.
