# Supplementary material for: Study of Bioengineered Zebra Fish Olfactory Receptor 131-2: Receptor Purification and Secondary Structure Analysis
Source: PLoS One. 2010 Nov 25;5(11):e15027. doi: 10.1371/journal.pone.0015027 (PMC2993934; doi:10.1371/journal.pone.0015027)
Supplement: Method S3 — Method for Supplementary Figure S3. Mass Spectrometry. (DOC) [file pone.0015027.s006.doc]

**Supplementary Method S3**

Mass Spectrometry

Purified OR131-2 protein was subjected to sodium dodecyl sulfate**-**polyacrylamide gel electrophoresis (SDS-PAGE) on Novex® 4-12% Bis Tris gels (Invitrogen, Singapore) and stained using Simply Blue (Invitrogen, Singapore). Protein gel bands were excised and digested with trypsin. The resulting peptide fragments were analyzed using LC-MS/MS by the Protein Mass Spectrometry service at the Biopolis Shared Facilities (Singapore). Proteolytic peptides were separated on a nanoAcquity C18 column, (1.7μm, 75μm ID x 150mm) (Waters, Milford, MA) on a nanoAcquity UPLC system (Waters, Milford, MA). Gradient elution with a water-acetonitrile-formic acid solvent system of peptides was carried out at a flow rate of 300 nL/min over 60 min. Electrospray mass spectra were acquired with a quadrupole time-of-flight mass spectrometer (Q-tof Premier, Waters, Milford, MA). An extensive blast search was performed on the identified peptide fragments using the bioinformatics program found on <http://blast.ncbi.nlm.nih.gov/Blast.cgi?PROGRAM=blastp&BLAST_PROGRAMS=blastp&PAGE_TYPE=BlastSearch&SHOW_DEFAULTS=on&LINK_LOC=blasthome>
